# COVID-Net CT-2: Enhanced Deep Neural Networks for Detection of COVID-19 From Chest CT Images Through Bigger, More Diverse Learning

**DOI:** 10.3389/fmed.2021.729287

**Published:** 2022-03-10

**Authors:** Hayden Gunraj, Ali Sabri, David Koff, Alexander Wong

**Affiliations:** ^1^Vision and Image Processing Lab, University of Waterloo, Waterloo, ON, Canada; ^2^Department of Radiology, McMaster University, Hamilton, ON, Canada; ^3^Niagara Health System, St. Catharines, ON, Canada; ^4^Hamilton Health Sciences, Hamilton, ON, Canada; ^5^Waterloo Artificial Intelligence Institute, University of Waterloo, Waterloo, ON, Canada; ^6^DarwinAI Corp., Waterloo, ON, Canada

**Keywords:** COVID-19, computed tomography, deep learning, image classification, radiology, SARS-CoV-2, pneumonia

## Abstract

The COVID-19 pandemic continues to rage on, with multiple waves causing substantial harm to health and economies around the world. Motivated by the use of computed tomography (CT) imaging at clinical institutes around the world as an effective complementary screening method to RT-PCR testing, we introduced COVID-Net CT, a deep neural network tailored for detection of COVID-19 cases from chest CT images, along with a large curated benchmark dataset comprising 1,489 patient cases as part of the open-source COVID-Net initiative. However, one potential limiting factor is restricted data quantity and diversity given the single nation patient cohort used in the study. To address this limitation, in this study we introduce enhanced deep neural networks for COVID-19 detection from chest CT images which are trained using a large, diverse, multinational patient cohort. We accomplish this through the introduction of two new CT benchmark datasets, the largest of which comprises a multinational cohort of 4,501 patients from at least 16 countries. To the best of our knowledge, this represents the largest, most diverse multinational cohort for COVID-19 CT images in open-access form. Additionally, we introduce a novel lightweight neural network architecture called COVID-Net CT S, which is significantly smaller and faster than the previously introduced COVID-Net CT architecture. We leverage explainability to investigate the decision-making behavior of the trained models and ensure that decisions are based on relevant indicators, with the results for select cases reviewed and reported on by two board-certified radiologists with over 10 and 30 years of experience, respectively. The best-performing deep neural network in this study achieved accuracy, COVID-19 sensitivity, positive predictive value, specificity, and negative predictive value of 99.0%/99.1%/98.0%/99.4%/99.7%, respectively. Moreover, explainability-driven performance validation shows consistency with radiologist interpretation by leveraging correct, clinically relevant critical factors. The results are promising and suggest the strong potential of deep neural networks as an effective tool for computer-aided COVID-19 assessment. While not a production-ready solution, we hope the open-source, open-access release of COVID-Net CT-2 and the associated benchmark datasets will continue to enable researchers, clinicians, and citizen data scientists alike to build upon them.

## 1. Introduction

The coronavirus disease 2019 (COVID-19) pandemic, caused by severe acute respiratory syndrome coronavirus 2 (SARS-CoV-2), continues to rage on, with multiple waves causing substantial harm to health and economies around the world. Real-time reverse transcription polymerase chain reaction (RT-PCR) testing remains the primary screening tool for COVID-19, where SARS-CoV-2 ribonucleic acid (RNA) is detected within an upper respiratory tract sputum sample (1). However, despite being highly specific, the sensitivity of RT-PCR can be relatively low (2, 3) and can vary greatly depending on the time since symptom onset as well as sampling method (3–5).

Clinical institutes around the world have explored the use of computed tomography (CT) imaging as an effective, complementary screening tool alongside RT-PCR (2, 5, 6). In particular, studies have shown CT to have great utility in detecting COVID-19 infections during routine CT examinations for non-COVID-19 related reasons such as elective surgical procedure monitoring and neurological examinations (7, 8). Other scenarios where CT imaging has been leveraged include cases where patients have worsening respiratory complications, as well as cases where patients with negative RT-PCR test results are suspected to be COVID-19 positive due to other factors. Early studies have shown that a number of potential indicators for COVID-19 infections may be present in chest CT images (2, 5, 6, 9–12), but may also be present in non-COVID-19 infections. This can lead to challenges for radiologists in distinguishing COVID-19 infections from non-COVID-19 infections using chest CT (13, 14).

Inspired by the potential of CT imaging as a complementary screening method and the challenges of CT interpretation for COVID-19 screening, we previously introduced COVID-Net CT (15), a convolutional neural network (CNN) tailored for detection of COVID-19 cases from chest CT images. We further introduced COVIDx CT, a large curated benchmark dataset comprising chest CT scans from a cohort of 1,489 patients derived from a collection by the China National Center for Bioinformation (CNCB) (16). Both COVID-Net CT and COVIDx CT were made publicly available as part of the COVID-Net (17, 18) initiative, an open-source initiative^1^ aimed at accelerating advancement and adoption of deep learning in the fight against the COVID-19 pandemic. While COVID-Net CT was able to achieve state-of-the-art COVID-19 detection performance, one potential limiting factor is the restricted quantity and diversity of CT imaging data used to learn the deep neural network given the entirely Chinese patient cohort used in the study. As such, a greater quantity and diversity in the patient cohort has the potential to improve generalization, particularly when COVID-Net CT is leveraged in different clinical settings around the world.

Motivated by the success and widespread adoption of COVID-Net CT and COVIDx CT, as well as their potential data quantity and diversity limitations, in this study we introduce COVID-Net CT-2, enhanced CNNs for COVID-19 detection from chest CT images which are trained using a large, diverse, multinational patient cohort. More specifically, we accomplish this through the introduction of two new CT benchmark datasets (COVIDx CT-2A and COVIDx CT-2B), the largest of which comprises a multinational cohort of 4,501 patients from at least 16 countries. To the best of the authors' knowledge, these benchmark datasets represent the largest, most diverse multinational cohorts for COVID-19 CT images available in open access form. Additionally, we introduce a novel lightweight neural network architecture called COVID-Net CT S, which is significantly smaller and faster than the previously introduced COVID-Net CT architecture and achieves an improved trade-off between performance and efficiency. Finally, we leverage explainability to investigate the decision-making behavior of COVID-Net CT-2 models to ensure decisions are based on relevant visual indicators in CT images, with the results for select patient cases being reviewed and reported on by two board-certified radiologists with 10 and 30 years of experience, respectively. The COVID-Net CT-2 networks and corresponding COVIDx CT-2 datasets are publicly available as part of the COVID-Net initiative (17, 18). While not a production-ready solution, we hope the open-source, open-access release of the COVID-Net CT-2 networks and the corresponding COVIDx CT-2 benchmark datasets will enable researchers, clinicians, and citizen data scientists alike to build upon them.

## 2. Materials and Methods

### 2.1. COVIDx CT-2 Benchmark Dataset

The original COVIDx CT benchmark dataset consists of chest CT scans collected by the China National Center for Bioinformation (CNCB) (16) which were carefully processed and selected to form a cohort of 1,489 patient cases. While COVIDx CT is significantly larger than many CT datasets for COVID-19 detection in literature, a potential limitation with leveraging COVIDx CT for training neural networks is the limited diversity in terms of patient demographics. More specifically, the cohort of patients used in COVIDx CT are collected in different provinces of China, and as such the characteristics of COVID-19 infection as observed in the chest CT images may not generalize to patients around the world outside of China. Therefore, increasing the quantity and diversity of the patient cohort in constructing new benchmark datasets could result in more diverse, well-rounded training of neural networks. In doing so, improved generalization and applicability for use in different clinical environments around the world can be achieved.

In this study, we carefully processed and curated CT images from several patient cohorts from around the world which were collected using a variety of CT equipment types, protocols, and levels of validation. By unifying CT imaging data from several cohorts from around the world, we created two diverse, large-scale benchmark datasets:

- **COVIDx CT-2A**: This benchmark dataset comprises 194,922 CT images from a multinational cohort of 3,745 patients between 0 and 93 years old (median age of 51) with strongly clinically-verified findings. The multinational cohort consists of patient cases collected by the following organizations and initiatives from around the world: (1) China National Center for Bioinformation (CNCB) (16) (China), (2) National Institutes of Health Intramural Targeted Anti-COVID-19 (ITAC) Program (hosted by TCIA (19), countries unknown), (3) Negin Radiology Medical Center (20) (Iran), (4) Union Hospital and Liyuan Hospital of Huazhong University of Science and Technology (21) (China), (5) COVID-19 CT Lung and Infection Segmentation initiative, annotated and verified by Nanjing Drum Tower Hospital (22) (Iran, Italy, Turkey, Ukraine, Belgium, some countries unknown), (6) Lung Image Database Consortium (LIDC) and Image Database Resource Initiative (IDRI) (23) (USA), and (7) Radiopaedia collection (24) (Iran, Italy, Australia, Afghanistan, Scotland, Lebanon, England, Algeria, Peru, Azerbaijan, some countries unknown).
- **COVIDx CT-2B**: This benchmark dataset comprises 201,103 CT images from a multinational cohort of 4,501 patients between 0 and 93 years old (median age of 51) with a mix of strongly verified findings and weakly verified findings. The patient cohort in COVIDx CT-2B consists of the multinational patient cohort we leveraged to construct COVIDx CT-2A, which have strongly clinically-verified findings, with additional patient cases with weakly verified findings collected by the Research and Practical Clinical Center of Diagnostics and Telemedicine Technologies, Department of Health Care of Moscow (MosMed) (25) (Russia). Notably, these additional cases are only included in the training dataset, and as such the validation and test datasets are identical to those of COVIDx CT-2A.

In both COVIDx CT-2 benchmark datasets, the findings for the chest CT volumes correspond to three different infection types: (1) novel coronavirus pneumonia due to SARS-CoV-2 viral infection (NCP), (2) common pneumonia (CP), and (3) normal controls. The image and patient distributions for the three infection types across training, validation, and test partitions are shown in Tables 1, 2 for COVIDx CT-2A and COVIDx CT-2B, respectively. Note that the data is partitioned at the patient level, and as such each patient appears in a single partition. For CT volumes labeled as NCP or CP, slices containing abnormalities were identified and assigned the same labels as the CT volumes. Notably, patient age was not available for all cases, and as such the age ranges and median ages reported above are based on patient cases for which age was available. The given range alludes to the inclusion of pediatric images in the COVIDx CT-2 datasets, which possess significant visual differences from adult images. However, we argue that leveraging COVID-19 pediatric cases may allow for the trained models to be more robust, and moreover only 26 of the included patients are under the age of 18. For images which were originally in Hounsfield units (HU), a standard lung window centered at −600 HU with a width of 1,500 HU was used to map the images to unsigned 8-bit integer range (i.e., 0–255).

**Table 1:** Distribution of chest CT slices and patient cases (in parentheses) by data partition and infection type in the COVIDx CT-2A dataset.

	**Infection type**			
**Partition**	**Normal**	**CP**	**NCP**	**Total**
Training	35,996 (321)	25,496 (558)	82,286 (1,958)	143,778 (2,837)
Validation	11,842 (126)	7,400 (190)	6,244 (166)	25,486 (482)
Test	12,245 (126)	7,395 (125)	6,018 (175)	25,658 (426)
Total	60,083 (573)	40,291 (873)	94,548 (2,299)	194,922 (3,745)

**Table 2:** Distribution of chest CT slices and patient cases (in parentheses) by data partition and infection type in the COVIDx CT-2B dataset.

	**Infection type**			
**Partition**	**Normal**	**CP**	**NCP**	**Total**
Training	35,996 (321)	25,496 (558)	88,467 (2,714)	149,959 (3,593)
Validation	11,842 (126)	7,400 (190)	6,244 (166)	25,486 (482)
Test	12,245 (126)	7,395 (125)	6,018 (175)	25,658 (426)
Total	60,083 (573)	40,291 (873)	100,729 (3,055)	201,103 (4,501)

**Figure 1:**
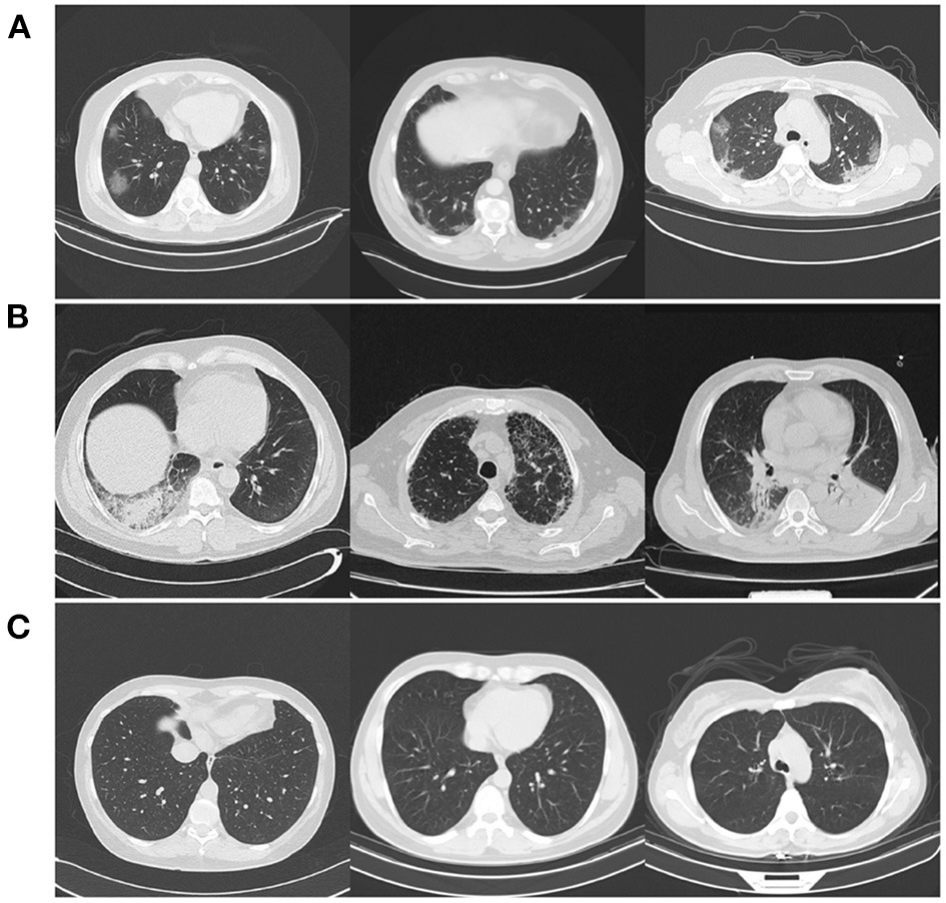
Example CT images from the COVIDx CT-2 benchmark datasets from each type of infection: **(A)** novel coronavirus pneumonia due to SARS-CoV-2 infection (NCP), **(B)** common pneumonia (CP), and **(C)** normal controls.

The rationale for creating two different COVIDx CT-2 benchmark datasets stems from the availability of weakly verified findings (i.e., findings not based on RT-PCR test results or final radiology reports), which can be useful for further increasing the quantity and diversity of patient cases that a neural network can be exposed to and can be of great interest for researchers, clinicians, and citizen scientists to explore and build upon while being made aware of the fact some of the CT scans do not have strongly verified findings available. Select patient cases from the benchmark datasets were reviewed and reported on by two board-certified radiologists with 10 and 30 years of experience, respectively. Both COVIDx CT-2A and COVIDx CT-2B benchmark datasets are publicly available^2^ as part of the COVID-Net initiative, with example CT images from each type of infection shown in Figure 1.

### 2.2. COVID-Net CT-2 Construction and Learning

**Figure 2:**
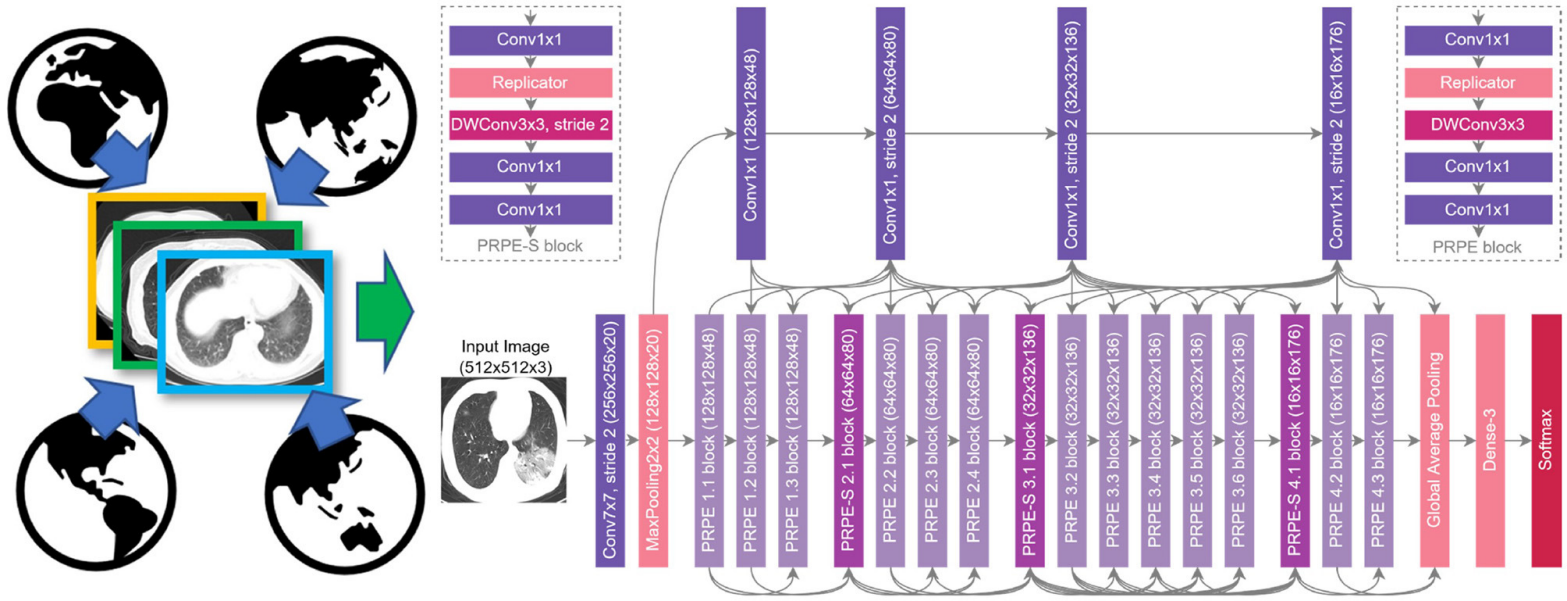
COVID-Net CT-2 S architecture design and COVIDx CT-2 benchmark. We leverage the COVID-Net CT network architecture (15) as the basis of the COVID-Net CT S network, which was discovered automatically via machine-driven design exploration.

By leveraging the COVIDx CT-2 benchmark datasets introduced in the previous section, we train a variety of CNNs in a way that better generalizes to a wide range of clinical scenarios. More specifically, six COVID-Net CT-2 deep neural networks are built based on six different deep CNN architecture designs: SqueezeNet (26), MobileNetV2 (27), EfficientNet-B0 (28), NASNet-A-Mobile (29), COVID-Net CT (15) (denoted COVID-Net CT L in this work), and a novel lightweight architecture called COVID-Net CT S. COVID-Net CT S shares the same macroarchitecture design as COVID-Net CT L, but with significantly more efficient microarchitecture designs. This greatly improved efficiency was achieved by leveraging machine-driven design exploration via generative synthesis (30), with the COVID-Net CT L architecture utilized as the initial design prototype. The COVID-Net CT S architecture is shown in Figure 2, and is made publicly available^3^.

The various COVID-Net CT-2 CNNs were trained on the COVIDx CT-2A dataset. For comparison purposes, we also trained each COVID-Net CT-2 network on the original COVIDx CT dataset (15) (referred to from here on as COVIDx CT-1 for clarity). To differentiate between networks trained on different datasets, we indicate CT-1 or CT-2 in the network names (e.g., MobileNetV2 CT-1, MobileNetV2 CT-2, COVID-Net CT-1 L, COVID-Net CT-2 L, etc.). Given the unsigned 8-bit integer format of the datasets, CT slices were normalized to the range [0, 1] through division by 255. Optimization was performed via stochastic gradient descent with momentum (31) using cross-entropy loss with L_2_ regularization, and the following hyperparameters were selected: learning rate = 5e-4, momentum = 0.9, λ_*L*_2__ = 1e-4, batch size = 8. To further increase data diversity beyond what is provided by the large multinational cohort, we leveraged random data augmentation in the form of cropping box jitter (±7.5%), rotation (±20^o^), horizontal and vertical shear (±0.2), horizontal flip, and intensity shift (±15 gray levels) and scaling (±10%), with all augmentations being applied with 50% probability. Training was stopped once the validation accuracy plateaued (i.e., early stopping). During training, we leveraged the batch re-balancing strategy used in (15) to ensure a balanced distribution of each infection type at the batch level.

We evaluate the various COVID-Net CT-2 networks using the COVIDx CT-2 test dataset. To assess performance, we report test accuracy as well as class-wise sensitivity, positive predictive value (PPV), specificity, and negative predictive value (NPV). Additionally, to assess the efficiency-performance trade-offs of the models, we report NetScore (32) which takes into account test accuracy, architectural complexity, as well as computational complexity within a unified metric. Qualitative evaluation through explainability was also performed, and is discussed in the next section.

### 2.3. Explainability-Driven Performance Validation

As with COVID-Net CT (15), we utilize GSInquire (33) to conduct explainability-driven performance validation. Using GSInquire, we audit the trained models to better understand and verify their decision-making behavior when analyzing CT images to predict the condition of a patient. This form of performance validation is particularly important in a clinical context, as the decisions made about patients' conditions can affect the health of patients via treatment and care decisions made using a model's predictions. Therefore, examining the decision-making behavior through model auditing is key to ensuring that the right visual indicators in the CT scans (e.g., ground-glass opacities) are leveraged for making a prediction as opposed to irrelevant visual cues (e.g., synthetic padding, artifacts, patient tables, etc.). Furthermore, incorporating interpretability in the validation process also increases the level of trust that a clinician has in leveraging such models for clinical decision support by adding an extra degree of algorithmic transparency.

To facilitate explainability-driven performance validation via model auditing, GSInquire provides an explanation of how a model makes a decision based on input data by identifying a set of critical factors within the input data that impact the decision-making process of the neural network in a quantitatively significant way. This is accomplished by probing the model with an input signal (in this case, a CT image) as the targeted stimulus signal and observing the reactionary response signals throughout the model, thus enabling quantitative insights to be derived through the inquisition process. These quantitative insights are then transformed and projected into the same space as the input signal to produce an interpretation (in this case, a set of critical factors in the CT image that quantitatively led to the prediction of the patient's condition). These interpretations can be visualized spatially relative to the CT images for greater insights into the decision-level behavior of COVID-Net CT-2. Compared to other explainability methods (34–38), this interesting nature of GSInquire in identifying quantitative impactful critical factors enables it to achieve explanations that better reflect the decision-making process of models (33). This makes it particularly suitable for quality assurance of models prior to clinical deployment to identify errors, biases, and anomalies that can lead to “right decisions for the wrong reasons.” The results obtained from GSInquire for select patient cases are further reviewed and reported on by two board-certified radiologists (AS and DK). The first radiologist (AS) has over 10 years of experience, while the second radiologist (DK) has over 30 years of radiology experience.

## 3. Results

### 3.1. Quantitative Analysis

To explore the efficacy of the COVID-Net CT-2 networks for COVID-19 detection from CT images, we conducted a quantitative evaluation of the trained networks using the COVIDx CT-2 test dataset. For comparison purposes, results for networks trained on the COVIDx CT-1 dataset (15) are also given. The training data used for each network is denoted by either CT-1 or CT-2 in the network name, as previously mentioned.

The test accuracy and NetScore (32) of each COVID-Net CT-2 network is shown in Table 3. It can be observed that all architectures achieve high test accuracy, with the best accuracy (99%) obtained using the MobileNetV2 and EfficientNet-B0 architectures trained on COVIDx CT-2A. Moreover, for all tested architectures, training on COVIDx CT-2A yields significant gains in test accuracy over training on COVIDx CT-1, with improvements ranging from +3.3 to +7.3% test accuracy. In terms of architectural and computational complexity, COVID-Net CT S possesses the fewest parameters (0.45 M) and floating-point operations (FLOPs, 1.94 G) while achieving an accuracy of 98.3%. The efficiency-performance trade-off of each model is assessed via its NetScore, of which COVID-Net CT-2 S' score of 83.3 is the highest and is significantly higher than the other tested networks. In contrast, the two COVID-Net CT-2 networks with the highest accuracies achieved significantly lower NetScores (74.1 and 70.7 for MobileNetV2 CT-2 and EfficientNet-B0 CT-2, respectively) owing to their significantly higher architectural and computational complexities. In resource-limited environments, balancing performance with efficiency is an important consideration.

**Table 3:** Comparison of parameters, FLOPs, accuracy (image-level), and NetScore (32) for the tested networks on the COVIDx CT-2 benchmark test dataset.

**Network**	**Parameters (M)**	**FLOPs (G)**	**Accuracy (%)**	**NetScore**
SqueezeNet CT-1 (26)	0.74	8.09	94.4	74.2
MobileNetV2 CT-1 (27)	2.23	3.33	91.7	72.8
EfficientNet-B0 CT-1 (28)	4.05	4.07	94.9	69.9
NASNet-A-Mobile CT-1 (29)	4.29	5.94	95.5	68.1
COVID-Net CT-1 L (15)	1.40	4.18	94.5	74.4
COVID-Net CT-1 S	**0.45**	**1.94**	93.2	82.4
SqueezeNet CT-2 (26)	0.74	8.09	98.7	75.0
MobileNetV2 CT-2 (27)	2.23	3.33	**99.0**	74.1
EfficientNet-B0 CT-2 (28)	4.05	4.07	**99.0**	70.7
NASNet-A-Mobile CT-2 (29)	4.29	5.94	98.8	68.7
COVID-Net CT-2 L (15)	1.40	4.18	98.4	75.1
COVID-Net CT-2 S	**0.45**	**1.94**	98.3	**83.3**

*Best results highlighted in bold*.

The sensitivity and PPV for each infection type on the COVIDx CT-2 test dataset is shown in Table 4. Examining the differences in sensitivity between COVID-Net CT-2 networks trained on COVIDx CT-1 and COVIDx CT-2A, it can be observed that significant gains in sensitivity are achieved through training on COVIDx CT-2A (+4.9 to +23.9%), with the best sensitivity (99.1%) obtained using the EfficientNet-B0 architecture. Notably, for the COVID-Net CT L and S architectures, increased sensitivity comes at the cost of a slight reduction in COVID-19 PPV, whereas for the other four architectures sensitivity and PPV are both improved. From a clinical perspective, high sensitivity ensures few false negatives which would lead to missed patients with COVID-19 infections, whereas high PPV ensures few false positives which add an unnecessary burden on the healthcare system, which is already stressed due to the ongoing pandemic.

**Table 4:** Sensitivity and positive predictive value (PPV) for each infection type at the image level on the COVIDx CT-2 benchmark test dataset.

	**Sensitivity (%)**			**PPV (%)**		
**Network**	**Normal**	**CP**	**NCP**	**Normal**	**CP**	**NCP**
SqueezeNet CT-1 (26)	92.9	98.3	92.8	97.5	96.6	86.3
MobileNetV2 CT-1 (27)	85.5	98.2	74.6	98.1	98.0	76.2
EfficientNet-B0 CT-1 (28)	99.3	97.8	82.5	94.8	93.4	97.6
NASNet-A-Mobile CT-1 (29)	98.9	97.9	85.5	96.0	94.6	95.5
COVID-Net CT-1 L (15)	98.8	99.0	80.2	96.1	90.2	97.6
COVID-Net CT-1 S	98.6	**99.2**	74.9	96.4	85.7	**98.4**
SqueezeNet CT-2 (26)	99.2	98.6	97.7	99.0	98.7	98.1
MobileNetV2 CT-2 (27)	99.3	98.9	98.5	**99.5**	99.0	98.0
EfficientNet-B0 CT-2 (28)	99.1	98.7	**99.1**	**99.5**	99.0	98.0
NASNet-A-Mobile CT-2 (29)	99.2	98.2	98.7	**99.5**	**99.3**	96.9
COVID-Net CT-2 L (15)	99.1	97.6	98.1	99.4	98.8	96.1
COVID-Net CT-2 S	**99.4**	99.1	97.3	99.3	98.3	96.3

*Best results highlighted in bold*.

The specificity and NPV for each infection type on the COVIDx CT-2 test dataset is shown in Table 5. Examining the differences in specificity between models trained on COVIDx CT-1 and COVIDx CT-2A, it can be observed that specificity does not change significantly in most cases, with a mix of improvements and reductions when switching from COVIDx CT-1 training to COVIDx CT-2 training. In contrast, when considering NPV, we observe consistent improvements ranging from no change (for COVID-Net CT L) to +6.4% (for EfficientNet-B0) when training on COVIDx CT-2A. The high specificity and NPV achieved by these models are important from a clinical perspective to ensure that COVID-19-negative predictions are indeed true negatives in the vast majority of cases, which facilitates rapid identification of COVID-19-negative patients.

**Table 5:** Specificity and negative predictive value (NPV) for each infection type at the image level on the COVIDx CT-2 benchmark test dataset.

	**Specificity (%)**			**NPV (%)**		
**Network**	**Normal**	**CP**	**NCP**	**Normal**	**CP**	**NCP**
SqueezeNet CT-1 (26)	97.8	98.6	95.5	93.8	99.3	97.7
MobileNetV2 CT-1 (27)	98.6	99.2	90.8	88.2	99.4	98.6
EfficientNet-B0 CT-1 (28)	95.0	97.2	99.4	99.3	99.1	94.9
NASNet-A-Mobile CT-1 (29)	96.2	97.7	98.8	99.0	99.1	95.7
COVID-Net CT-1 L (15)	99.4	99.5	98.8	99.2	99.0	99.4
COVID-Net CT-1 S	96.6	93.3	**99.6**	98.7	**99.6**	92.8
SqueezeNet CT-2 (26)	99.1	99.5	99.4	99.3	99.4	99.3
MobileNetV2 CT-2 (27)	99.5	99.6	99.4	**99.4**	**99.6**	99.5
EfficientNet-B0 CT-2 (28)	**99.6**	99.6	99.4	99.2	99.5	**99.7**
NASNet-A-Mobile CT-2 (29)	99.5	**99.7**	99.0	99.3	99.3	99.6
COVID-Net CT-2 L (15)	99.4	99.5	99.8	99.2	99.0	99.4
COVID-Net CT-2 S	99.4	99.3	98.8	98.4	**99.6**	99.2

*Best results highlighted in bold*.

Given the diverse nature of the COVIDx CT-2 test dataset, these experimental results are particularly promising in terms of network generalization and applicability for use in different clinical environments. Additionally, the reduced performance observed when COVIDx CT-1 is used for training illustrates the value of larger, diverse training data for improving model performance in a variety of clinical scenarios.

### 3.2. Qualitative Analysis

**Figure 3:**
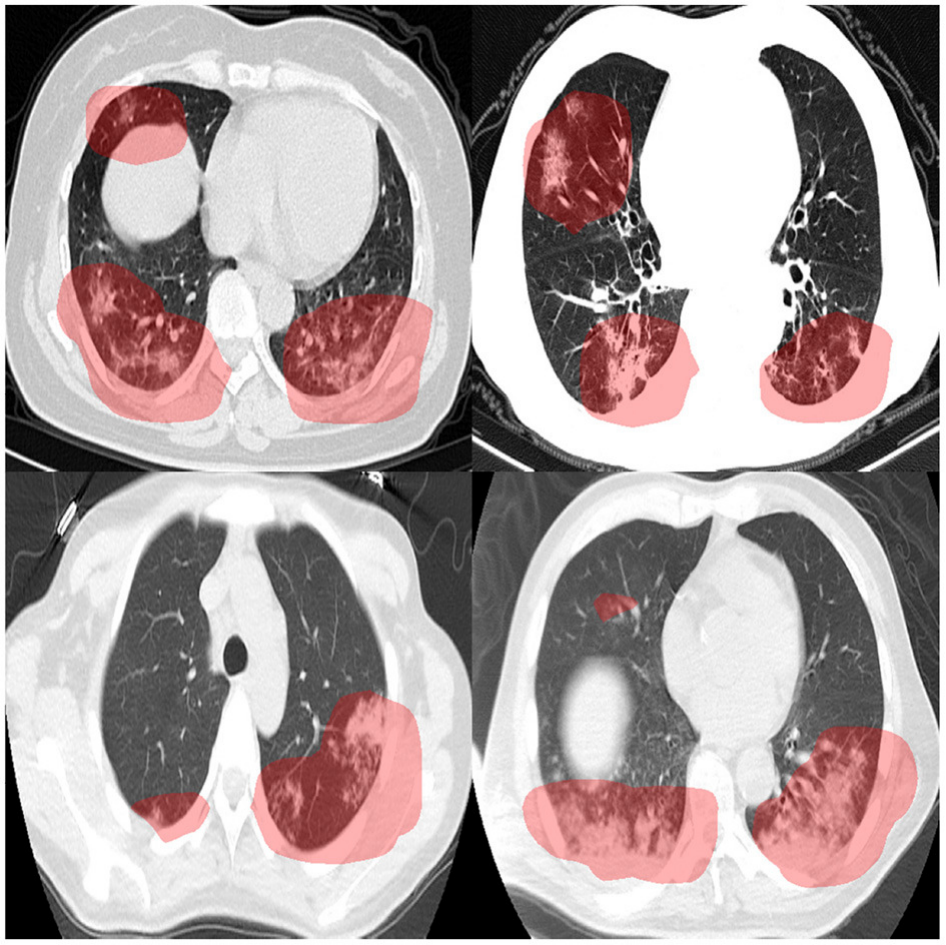
Example chest CT images from four COVID-19 cases reviewed and reported on by two board-certified radiologists, and the associated critical factors (highlighted in red) as identified by GSInquire (33) for COVID-Net CT-2 L. Based on the observations made by two expert radiologists, it was found that the critical factors leveraged by COVID-Net CT-2 L are consistent with radiologist interpretation.

**Figure 4:**
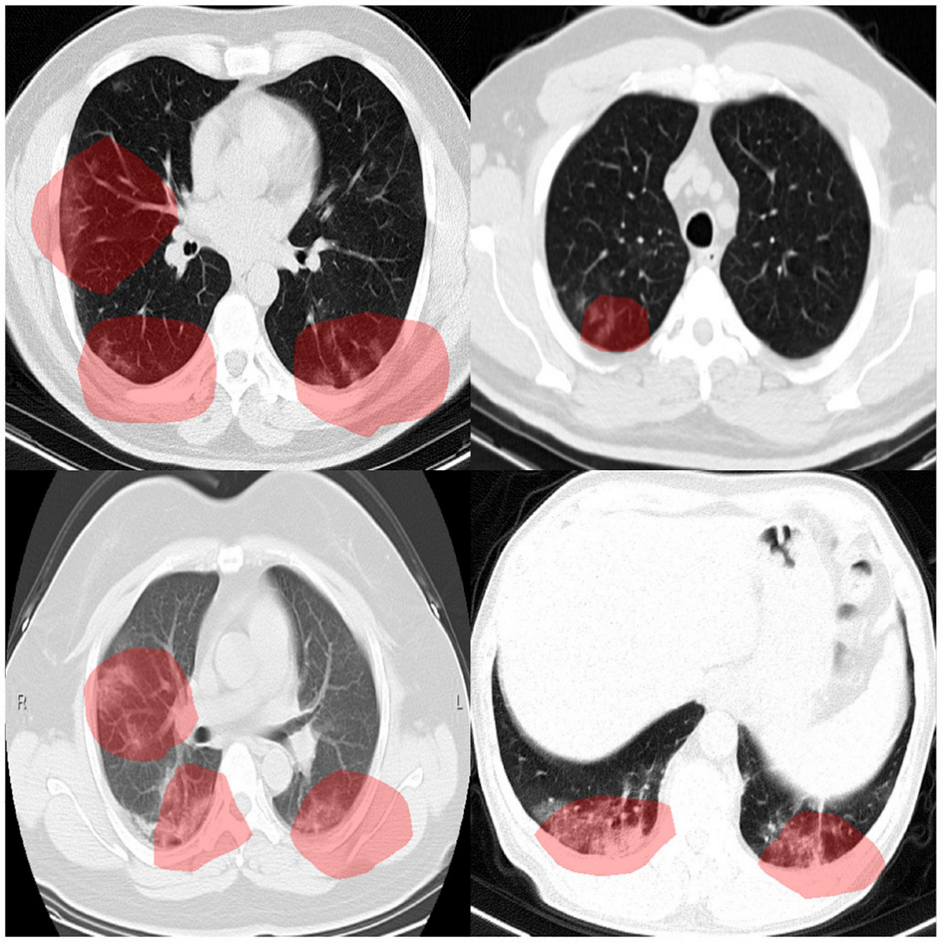
Example chest CT images from four COVID-19 cases, and the associated critical factors (highlighted in red) as identified by GSInquire (33) for COVID-Net CT-2 S.

To audit the decision-making behavior of COVID-Net CT-2 and ensure that it is leveraging relevant visual indicators when predicting the condition of a patient, we conducted explainability-driven performance validation using the COVIDx CT-2 benchmark test dataset, and the results obtained using COVID-Net CT-2 L for select patient cases are further reviewed and reported on by two board-certified radiologists. The critical factors identified by GSInquire for example chest CT images from the four COVID-19-positive cases that were reviewed are shown in Figure 3, and additional examples for COVID-Net CT-2 S are shown in Figure 4.

Overall, it can be observed from the GSInquire-generated visual explanations that both COVID-Net CT-2 L and COVID-Net CT-2 S are mainly utilizing visible lung abnormalities to distinguish between COVID-19-positive and COVID-19-negative cases. As such, this auditing process allows us to determine that COVID-Net CT-2 is indeed leveraging relevant visual indicators in the decision-making process as opposed to irrelevant visual indicators such as imaging artifacts, artificial padding, and patient tables. This performance validation process also reinforces the importance of utilizing explainability methods to confirm proper decision-making behavior in neural networks designed for clinical decision support.

### 3.3. Radiologist Findings

The expert radiologist findings and observations with regards to the critical factors identified by GSInquire for each of the four patient cases shown in Figure 3 are as follows. In all four cases, COVID-Net CT-2 L detected them to be novel coronavirus pneumonia due to SARS-CoV-2 viral infection, which was clinically confirmed.

**Case 1 (top-left of** Figure 3**)**. It was observed by one of the radiologists that there are bilateral peripheral mixed ground-glass and patchy opacities with subpleural sparing, which is consistent with the identified critical factors leveraged by COVID-Net CT-2 L. The absence of large lymph nodes and effusion further helped the radiologist point to novel coronavirus pneumonia due to SARS-CoV-2 viral infection. The degree of severity is observed to be moderate to high. It was confirmed by the second radiologist that the identified critical factors leveraged by COVID-Net CT-2 L are correct areas of concern and represent areas of consolidation with a geographic distribution that is in favor of novel coronavirus pneumonia due to SARS-CoV-2 viral infection.

**Case 2 (top-right of** Figure 3**)**. It was observed by one of the radiologists that there are bilateral peripherally-located ground-glass opacities with subpleural sparing, which is consistent with the identified critical factors leveraged by COVID-Net CT-2 L. As in Case 1, the absence of large lymph nodes and large effusion further helped the radiologist point to novel coronavirus pneumonia due to SARS-CoV-2 viral infection. The degree of severity is observed to be moderate to high. It was confirmed by the second radiologist that the identified critical factors leveraged by COVID-Net CT-2 L are correct areas of concern and represent areas of consolidation with a geographic distribution that is in favor of novel coronavirus pneumonia due to SARS-CoV-2 viral infection.

**Case 3 (bottom-left of** Figure 3**)**. It was observed by one of the radiologists that there are peripheral bilateral patchy opacities, which is consistent with the identified critical factors leveraged by COVID-Net CT-2 L. Unlike the first two cases, there is small right effusion. However, as in Cases 1 and 2, the absence of large effusion further helped the radiologist point to novel coronavirus pneumonia due to SARS-CoV-2 viral infection. Considering that the opacities are at the base, a differential of atelectasis change was also provided. The degree of severity is observed to be moderate. It was confirmed by the second radiologist that the identified critical factors leveraged by COVID-Net CT-2 L are correct areas of concern and represent areas of consolidation.

**Case 4 (bottom-right of** Figure 3**)**. It was observed by one of the radiologists that there are peripherally located asymmetrical bilateral patchy opacities, which is consistent with the identified critical factors leveraged by COVID-Net CT-2 L. As in Cases 1 and 2, the absence of lymph nodes and large effusion further helped the radiologist point to novel coronavirus pneumonia due to SARS-CoV-2 viral infection, but a differential of bacterial pneumonia was also provided considering the bronchovascular distribution of patchy opacities. In addition, there is no subpleural sparing. This highlights the potential difficulties in differentiating between novel coronavirus pneumonia and common pneumonia. It was confirmed by the second of the radiologists that the identified critical factors leveraged by COVID-Net CT-2 L are correct areas of concern and represent areas of consolidation with a geographic distribution that is in favor of novel coronavirus pneumonia due to SARS-CoV-2 viral infection.

Therefore, it can be observed that the explainability-driven validation process shows consistency between the decision-making process of COVID-Net CT-2 and radiologist interpretation, which suggests strong potential for computer-aided COVID-19 assessment within a clinical environment.

## 4. Discussion

In this work, we introduced COVID-Net CT-2, enhanced CNNs tailored for the purpose of COVID-19 detection from chest CT images. Two new CT benchmark datasets were introduced and used to facilitate the training of COVID-Net CT-2, and these datasets represent the largest, most diverse multinational cohorts of their kind available in open-access form, spanning cases from at least 16 countries. Experimental results show that the COVID-Net CT-2 networks are capable of not only achieving strong quantitative results, but also doing so in a manner that is consistent with radiologist interpretation via explainability-driven performance validation. The results are promising and suggest the strong potential of neural networks as an effective tool for computer-aided COVID-19 assessment.

Given the severity of the COVID-19 pandemic and the potential for deep learning to facilitate computer-assisted COVID-19 clinical decision support, a number of deep learning systems have been proposed in research literature for detecting SARS-CoV-2 infections using CT images (14–16, 21, 39–50), with a comprehensive review performed by Islam et al. (51). While some proposed deep learning systems focus on binary detection (SARS-CoV-2 positive vs. negative) (50), several proposed systems operate at a finer level of granularity by further identifying whether SARS-CoV-2-negative cases are normal control (16, 39, 47, 48), SARS-CoV-2 negative pneumonia [e.g., bacterial pneumonia, viral pneumonia, community-acquired pneumonia (CAP), etc.] (16, 39–42, 48, 49), or non-pneumonia (41).

The majority of the proposed deep learning systems for COVID-19 detection from CT images rely on pre-existing network architectures that were designed for other image classification tasks. A large number of proposed systems additionally rely on segmentation of the lung region and/or lung lesions (14, 16, 39–41, 44, 45, 47, 48). Some proposed systems also modify pre-existing network architectures; for example, Xu et al. (39) add location-attention modules to a ResNet-18 (52) backbone architecture, and Li et al. (41) and Bai et al. (40) add pooling operations to 2D architectures for volume-driven detection. Of the deep learning systems that proposed new neural network architectures, Shah et al. (43) proposed a 10-layer CNN architecture named CTnet-10, which ultimately showed lower detection performance than pre-existing architectures in literature. Zheng et al. (45) proposed a 3D CNN architecture named DeCovNet which is capable of volume-driven detection. Gunraj et al. (15) used machine-driven design to construct a tailored architecture, which was found to outperform three existing architectures. Hasan et al. (53) combined features obtained from a Q-deformed entropy model and custom CNN and used them to train a long short-term memory-based classifier. This feature fusion approach was found to outperform either of the feature sets alone. Finally, Javaheri et al. (54) use a U-Net-based architecture known as BCDU-Net to pre-process CT images before performing classification using a custom CNN architecture. The proposed BCDU-Net was trained using artificial data based on control cases and the 3D CNN classifier was then trained to classify CT volumes as COVID-19, community-acquired pneumonia, or control.

While the concept of leveraging deep learning for COVID-19 detection from CT images has been previously explored, even the largest studies in research literature have been limited in terms of quantity and/or diversity of patients, with many limited to single-nation cohorts (14–16, 21, 39, 42, 54, 55). For example, the studies by Mei et al. (14), Gunraj et al. (15), Ning et al. (21), and Zhang et al. (16) were all limited to Chinese patient cohorts consisting of 905 patients, 1,489 patients, 1,521 patients, and 3,777 patients, respectively. Moreover, the studies by Ardakani et al. (42) and Javaheri et al. (54) leveraged patient cohorts from Iran including 108 and 335 patients, respectively. Multinational patient cohorts have been leveraged in several studies, but have typically been limited to few patients or few countries. For example, Hasan et al. (53) leveraged a multinational cohort (countries unknown) of 321 patients, Harmon et al. (50) leveraged a cohort of 2,617 patients across four countries, and Jin et al. (47) leveraged a cohort of 9,025 patients across at least three countries. To the best of the authors' knowledge, the multinational patient cohort introduced in this study represents the most diverse multinational patient cohort at 4,501 patients across at least 16 countries, and is the largest available in open-access form. By building the COVID-Net CT-2 deep neural networks using a large multinational patient cohort, we can better study the generalization capabilities and applicability of deep learning for computer-assisted assessment in a wide variety of clinical scenarios and demographics.

With the tremendous burden the ongoing COVID-19 pandemic has put on healthcare systems and healthcare workers around the world, the hope is that research such as COVID-Net CT-2 and open-source initiatives such as the COVID-Net initiative can accelerate the advancement and adoption of deep learning solutions within a clinical setting to aid front-line health workers and healthcare systems in improving clinical workflow efficiency and effectiveness in the fight against the COVID-19 pandemic. While to the best of the authors' knowledge this research does not put anyone at a disadvantage, it is important to note that COVID-Net CT-2 is not a production-ready solution and is meant for research purposes. As such, predictions made by COVID-Net CT-2 should not be utilized blindly and should instead be built upon and leveraged in a human-in-the-loop fashion by researchers, clinicians, and citizen data scientists alike. Future work involves leveraging the pre-trained networks for downstream tasks such as lung function prediction, severity assessment, and actionable predictions for guiding personalized treatment and care for SARS-CoV-2 positive patients.

## Data Availability Statement

Publicly available datasets were analyzed in this study. This data can be found at: https://www.kaggle.com/hgunraj/covidxct.

## Ethics Statement

This study was reviewed and approved by the University of Waterloo Ethics Board (42235). Written informed consent from the participants or their legal guardian/next of kin was not required to participate in this study in accordance with national legislation and institutional requirements.

## Author Contributions

HG and AW conceived the experiments. HG conducted the experiments. DK and AS reviewed and reported on select patient cases and corresponding explainability results. All authors analyzed the results and reviewed the manuscript.

## Conflict of Interest

AW was affiliated with DarwinAI Corp. DarwinAI Corp. provided computing support for this work, specifically through access to their deep learning development platform. The remaining authors declare that the research was conducted in the absence of any commercial or financial relationships that could be construed as a potential conflict of interest.

## Publisher's Note

All claims expressed in this article are solely those of the authors and do not necessarily represent those of their affiliated organizations, or those of the publisher, the editors and the reviewers. Any product that may be evaluated in this article, or claim that may be made by its manufacturer, is not guaranteed or endorsed by the publisher.

## Footnotes

^1^ https://alexswong.github.io/COVID-Net

^2^ https://www.kaggle.com/hgunraj/covidxct

^3^ https://github.com/haydengunraj/COVIDNet-CT
